# Reversible formation of tetraphenylpentalene, a room temperature stable antiaromatic hydrocarbon†

**DOI:** 10.1039/d4sc06439a

**Published:** 2024-11-25

**Authors:** Hugh J. Sanderson, Andreas Helbig, Gabriele Kociok-Köhn, Holger Helten, Ulrich Hintermair

**Affiliations:** a Department of Chemistry and Institute for Sustainability, University of Bath Claverton Down Bath BA2 7AY UK u.hintermair@bath.ac.uk; b Julius-Maximilians-Universität Würzburg, Institute of Inorganic Chemistry and Institute for Sustainable Chemistry & Catalysis with Boron Am Hubland 97074 Würzburg Germany holger.helten@uni-wuerzburg.de; c Chemical Characterisation Facility, University of Bath Claverton Down Bath BA2 7AY UK

## Abstract

1,3,4,6-Tetraphenylpentalene (Ph_4_Pn) has been synthesised by chemical oxidation of the corresponding pentalenide complex Mg[Ph_4_Pn] with iodine. Ph_4_Pn is a rare example of a room-temperature stable hydrocarbon that is antiaromatic by Hückel's rule and has been fully characterised by NMR and UV-vis spectroscopy, mass spectrometry as well as single-crystal X-ray diffraction. Quantum chemical studies including nucleus-independent chemical shift (NICS) and anisotropy of the induced current density (ACID) calculations showed the existence of an 8π antiaromatic core decorated with four independent 6π aromatic substituents. The formation of Ph_4_Pn is reversible and it can be reduced back to the 10π aromatic Ph_4_Pn^2−^ with potassium.

## Abstract 2

A rare example of a small, easily synthesisable hydrocarbon which can reversibly switch between aromatic and anti-aromatic states without any skeletal or conformational changes is reported.

## Graphical abstract

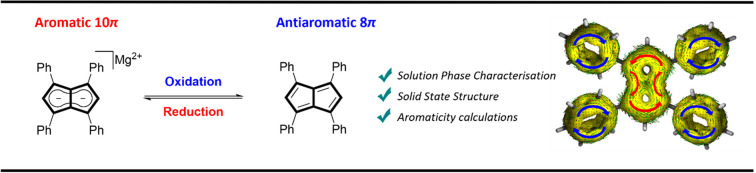

## 1. Introduction

Aromaticity, and by extension antiaromaticity, are core concepts in chemistry with different classifications.^1^ The most familiar is Hückel aromaticity, embodied by the 4*n* + 2 π-electron rule developed by Doering and Detert,^2^ which is used for planar closed-shell monocyclic conjugated systems.^3^ The opposite, Hückel antiaromaticity, is termed for molecules that have a 4*n* π-electron count yet satisfy the rest of the criteria.^4^ Whereas aromaticity serves to provide a molecule with increased stability, antiaromaticity imparts destabilisation to the molecule along with other contrasting spectroscopic and magnetic properties (such as paratropic ring currents).^5,6^ The concept of (anti)aromaticity was later extended to open-shell systems by Baird,^7^ who showed that molecules with a 4*n* π-electron count display aromaticity in the triplet state whilst those with a formal 4*n* + 2 π-electron count exhibit antiaromatic behaviour in the triplet state.^8^ Other types of (anti)aromaticity have also been reported,^1^ and mechanisms for electron delocalisation are known beyond π-conjugation.^9–13^

Compared to the interest in the different forms of aromaticity,^5^ less attention has been paid to antiaromatic molecules and the properties they can possess. Hückel antiaromaticity can result in a lowering of the HOMO–LUMO gap^14^ and the presence of a low-lying triplet state which may exhibit Baird aromaticity.^4,7,8,15,16^ Thus, molecules possessing antiaromaticity have found use in areas such as optoelectronics, for example.^17–20^ However, the destabilisation that arises from a fully conjugated 4*n* π-electron system often results in highly unstable molecules that react readily to relieve the antiaromatic strain – *e.g.* through dimerisation or by adoption of non-planar conformations.^21^ The idea of concealed antiaromaticity has been proposed as a framework with which to identify the structural motifs used to stabilise antiaromatic systems, such that upon oxidation/reduction or photoexcitation aromatic systems may be formed.^22^ Such motifs presented include sharing of 2π or 4π-electron fragments by a locally aromatic group to the 4*n* π-system and internal connections (*e.g.* C–C bonds) within the 4*n* π-system itself.

**Scheme 1:**
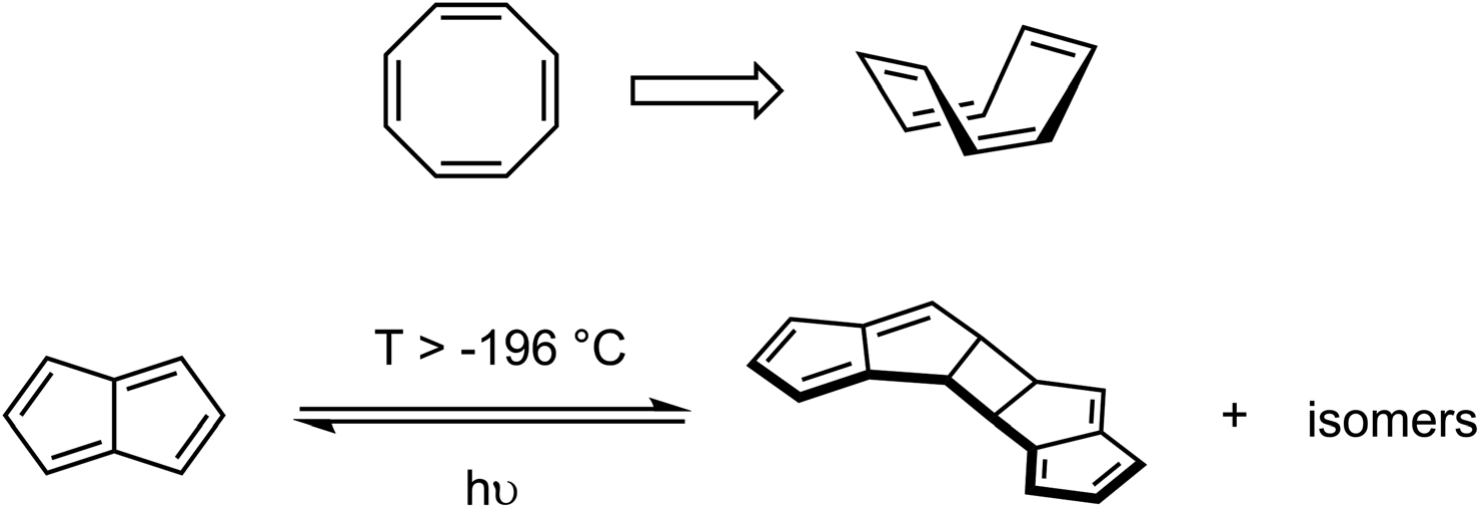
Examples of carbocyclic 8π systems: non-planar conformation of COT (top) and dimerization of Pn (bottom).

A classic example of a 4*n* π-system that alleviates, or conceals, its antiaromaticity is cyclooctatetraene (C_8_H_8_, COT) which adopts a non-planar boat conformation thus avoiding π-conjugation and formation of antiaromatic ring currents (Scheme 1). Pentalene (C_8_H_6_, Pn) is an 8 π-electron hydrocarbon related to COT*via* a transannular ring closure^23^ that forces it to adopt a coplanar structure and thus yields a Hückel antiaromatic system. As a consequence Pn dimerises *via* a [2 + 2] cycloaddition pathway (*c.f.* cyclobutadiene) above −196 °C to form a non-aromatic pentafulvene-type system.^24,25^ This process indicates the presence of a low-lying triplet state since such a reaction is believed to proceed *via* diradical intermediates.^26^ The increased rigidity imparted by the transannular bond also prevents conformational rearrangements that are often seen when switching between aromatic and antiaromatic states of larger molecules.^27–34^ However, to date no crystallographic data of a pentalene and its corresponding pentalenide have been reported to compare the extent of any changes.

The most common method to stabilise (or conceal) the antiaromatic character of pentalene is through annelation with other π systems such as in benzopentalenes, with the first dibenzopentalene reported in 1912, nearly 50 years prior to the first report of an isolable Pn.^35,36^ Conjugation of the pentalene to locally aromatic benzene groups imparts stability onto the antiaromatic core and renders dibenzopentalenes to be bench-stable despite being formally Hückel antiaromatic 4*n* π-electron systems.^16,18,37–39^ Annelation can also be used to vary the degree of (anti)aromaticity, as shown for dinaphtho[2,1,*a*,*f*]pentalene,^40^ an effect which is useful for tuning the electronics of polycyclic pentalene derivatives for potential use in organic semiconductors.^41–43^

**Fig. 1:**
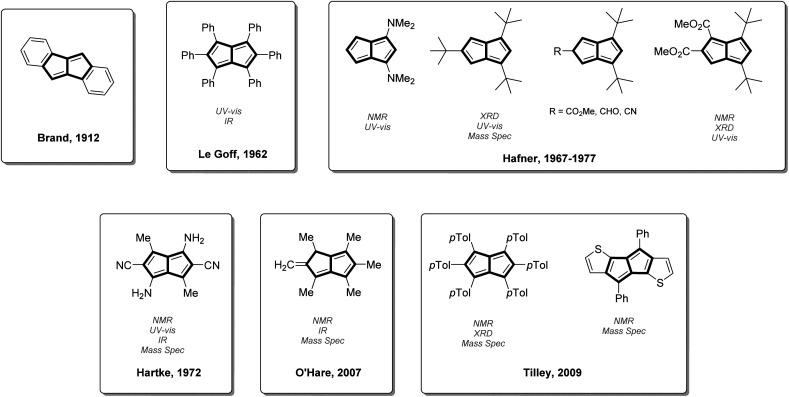
Previously reported stable pentalene derivatives and their means of characterisation.

However, reports of stable Pn derivatives that do not rely on annelation with aromatic groups are sparse (Fig. 1).^44–50^ 1,3,5-^*t*^Bu_3_Pn was the first Pn derivative to be characterised by single crystal XRD which confirmed the coplanarity of the two rings in the Pn core and showed bond alternation consistent with localised C=C bonds.^51^ The stabilisation of 1,3,5-^*t*^Bu_3_Pn arises from the steric bulk of the ^*t*^Bu substituents providing a high kinetic barrier that prevents the [2 + 2] cycloaddition from occurring; the less sterically hindered derivatives 1,3-^*t*^Bu_2_-5-RPn (R = CO_2_Me, CHO or CN) were found to exist in equilibrium with their [2 + 2] dimers.^49^ 1,2,3,4,5,6-hexamethylpentalene (Pn*) avoids forming an antiaromatic π-system through an exocyclic C=C bond which results in the Pn core not being fully sp^2^ hybridised.^44^ The corresponding [2 + 2] dimer could be accessed through oxidation of the dianion Pn*^2−^ by FeCl_2_.^52^ Another hexasubstituted Pn, an aminopentalenecarbonitrile, was reported by Hartke where the opposing push–pull effects of the amino and nitrile groups were proposed to electronically stabilise the antiaromatic core.^45^ A thienylpentalene has been reported where the aromatic heterocycle stabilises the antiaromaticity of the core as in benzopentalenes. In the same report a hexaarylated Pn derivative bearing *p*-tolyl substituents was described.^50^ The hexasubstitution pattern is likely to impart a significant steric barrier to the [2 + 2] dimerisation, however, the electronic influence of the aromatic tolyl-substituents on the Pn core is unknown. More recently a tetra-substituted Pn with cyclopropyl groups was described, however, the cyclo-propyl groups did not impart enough steric or electronic stabilisation to prevent [2 + 2] dimerisation.^53^ Le Goff reported the synthesis of hexaphenylpentalene from the base-catalysed condensation of a cyclopentadiene with an α,β-unsaturated carbonyl compound followed by oxidation of the resultant dihydropentalene.^36^ Whilst the synthetic rationale of forming substituted PnH_2_ from cyclopentadienes and α,β-unsaturated carbonyls is a well-established pathway,^54–58^ the subsequent oxidation of PnH_2_ to Pn is less well known. Le Goff also noted that a sample of Li_2_[Ph_6_Pn] could be oxidised by addition of I_2_ to yield Ph_6_Pn,^36^ a route that in theory should allow facile formation of Pn derivatives where the only by-product would be an inorganic salt. Recently we reported the synthesis of the first alkaline earth pentalenide Mg[Ph_4_Pn].^59^ Herein we report the remarkably facile and fully reversible oxidation of Mg[Ph_4_Pn] to Ph_4_Pn which has been fully characterised by NMR and UV-vis spectroscopy as well as X-ray diffraction. The antiaromaticity of the Pn core and the steric and electronic influence of the phenyl substituents have been investigated by computational studies.

## 2. Results and discussion

**Fig. 2:**
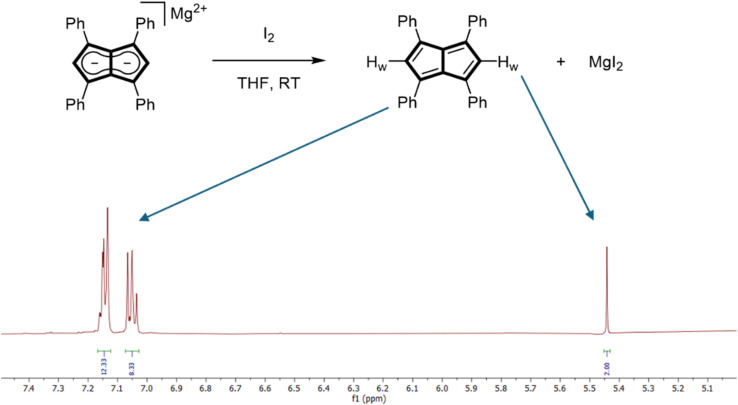
Synthesis of 1,3,4,6-Ph_4_Pn (top) and its 500 MHz room temperature ^1^H NMR spectrum in THF-H_8_ (bottom).

The addition of one equivalent of iodine to a THF solution of Mg[Ph_4_Pn] at room temperature led to an immediate colour change from bright orange to dark yellow, with a precipitate of MgI_2_ forming after a few minutes. NMR spectroscopic analysis of the filtered solution showed the complete and selective formation of Ph_4_Pn (Fig. 2). When either an excess of iodine or an equivalent of bromine was used instead, an unidentifiable mixture of products originating from unselective (over)oxidation was observed. This difference in reactivity can be attributed to the increased oxidation power of Br_2_ over I_2_ (*E*^0^ = 1.07 V for Br_2_; *E*^0^ = 0.54 V for I_2_)^60^ and its propensity to undergo bromination reactions. Previous attempts to oxidise Ph_4_PnH_2_ or Ph_4_Pn^2−^ to Ph_4_Pn with *N*-bromosuccinimide were unsuccessful,^61^ although Cu(ii), which has a lower oxidation potential (*E*^0^ = 0.34 V) than iodine, has previously been reported to facilitate the oxidative coupling of Li_2_[Pn] to afford [Pn]_2_.^25,62^

The room temperature ^1^H NMR spectrum of Ph_4_Pn showed a *C*_2h_ symmetrical molecule with three well-resolved, diamagnetic signals observed at 7.14, 7.05 and 5.44 ppm (Fig. 2). The chemical shift of the two equivalent wingtip protons H_w_ in Ph_4_Pn was found to be 1.5 ppm upfield relative to Ph_4_Pn^2−^ (Table 1), and the corresponding wingtip carbons C_w_ had a resonance at 134.9 ppm, a 20 ppm downfield shift relative to the respective signal in Ph_4_Pn^2−^. These shifts are consistent with formation of a localised olefinic π-system arising from an 8π antiaromatic pentalene and indicate full oxidation of the core of Ph_4_Pn^2−^ to Ph_4_Pn, with the four equivalent phenyl groups remaining largely unperturbed. The cleanliness of the crude ^1^H NMR spectrum also indicates that in solution there is no significant [2 + 2] dimer formation. Variable temperature NMR studies from −80 °C to 60 °C in THF showed no discernible changes (Fig. S4†), indicating monomeric Ph_4_Pn to be remarkably stable under inert conditions, although exposure to air quickly led to decomposition.

**Table 1:** Key analytical features of Ph_4_PnH_2_, Mg[Ph_4_Pn], Ph_4_Pn and [Ph_4_Pn]_2_

	*δ* ^1^H_w_^a^ (^13^C_w_)/ppm	C–C perimeter/Å	C–C bridge/Å	Aryl twist angle/°
Ph_4_PnH_2_	7.45 (139.4)	1.357(6)–1.512(8)	1.448(7)	43.3–47.0
Mg[Ph_4_Pn]	6.80 (115.5)	1.406(3)–1.455(3)	1.451(3)	17.1–35.2
Ph_4_Pn	5.42 (134.9)	1.388(2)–1.464(3)	1.450(3)	31.7–42.3
[Ph_4_Pn]_2_	n/a	1.357(2)–1.517(2)	1.471(2), 1.476(2)	11.9–47.3

a Data for Ph_4_PnH_2_ in acetone-d_6_, Mg[Ph_4_Pn] and Ph_4_Pn in THF-H_8_.

**Fig. 3:**
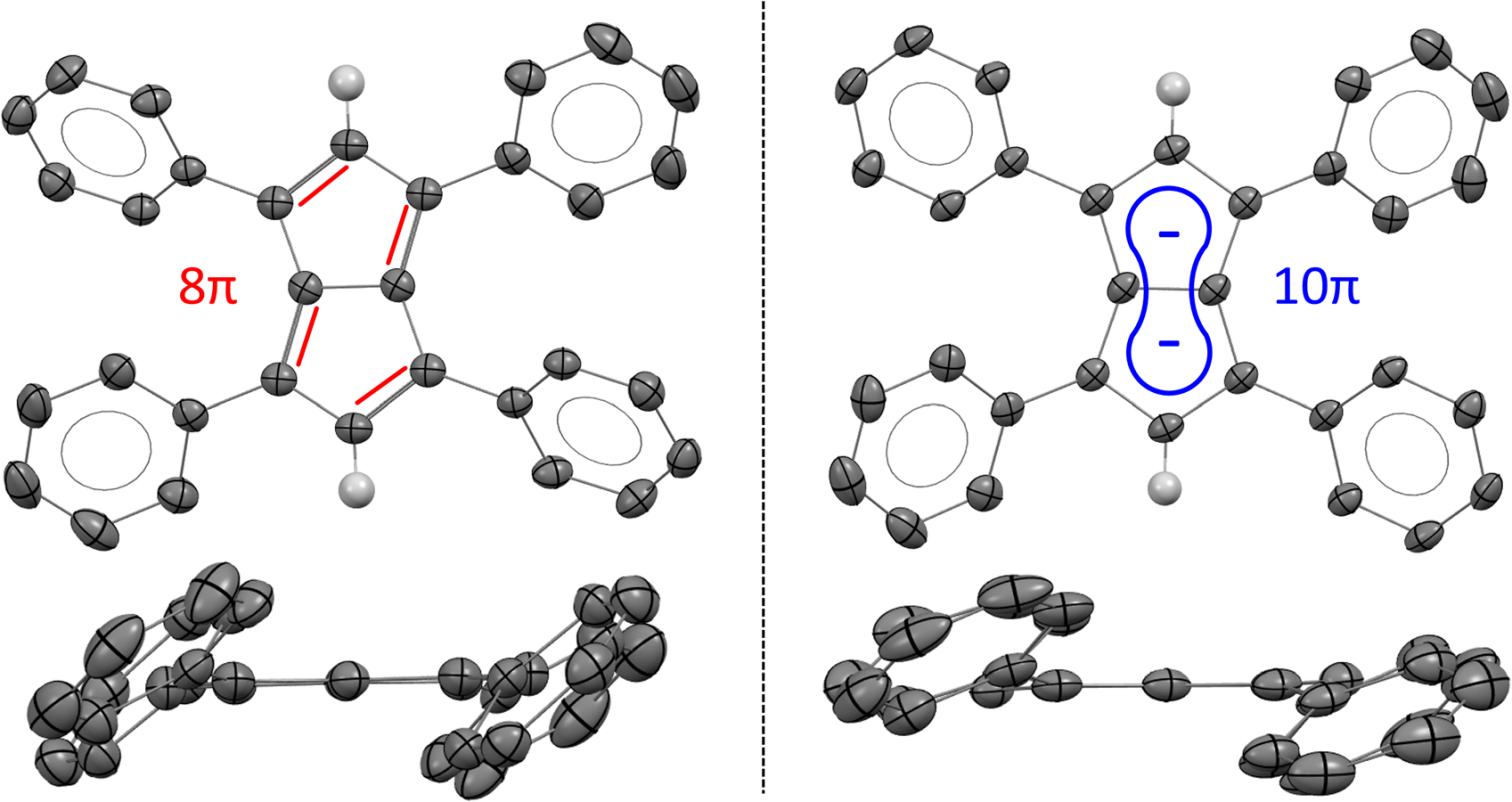
X-ray crystal structures of 1,3,4,6-Ph_4_Pn (left; top and side views) and 1,3,4,6-Ph_4_Pn^2−^ (right; top and side views)^63^ with thermal ellipsoids at the 50% probability level. Phenyl hydrogen atoms omitted for clarity, and double bonds and aromatic conjugation added for illustration.

XRD analysis of single crystals grown from THF at −35 °C confirmed the structure of Ph_4_Pn with retention of a bicyclic, co-planar Pn core (Fig. 3). Bond alternation within the sp^2^ perimeter was evident by C–C distances within the Pn ring ranging from 1.388(2)–1.464(3) Å. For comparison, the average C–C bond length in Mg[Ph_4_Pn] was found to range between 1.406(3)–1.455(3) Å (Table 1).^59^ The C=C bond lengths in Ph_4_Pn were longer than in the non-aromatic Ph_4_PnH_2_ (1.36(1)–1.37(1) Å)^57^ in reflection of its antiaromatic character. The C–C bridgehead bond length of 1.450(3) Å was identical to the equivalent distance in Mg[Ph_4_Pn] (1.451(3) Å), suggesting the electronics of the Pn core do not significantly influence this bond. The C–C/C=C bond alternation found in Ph_4_Pn was less pronounced than in 1,3,5-^t^Bu_3_Pn where the C–C bonds ranged from 1.46–1.54 Å and the C=C bonds between 1.28–1.41 Å, which is likely a consequence of the different electronic nature of the substituents on the pentalene core (alkyl *versus* aryl).^51^ No stacking of Ph_4_Pn was observed in the solid or solution state, ruling out the formation of a so-called “3D aromatic” system as a means of alleviating the antiaromatic 8π count.^64–66^

As in Mg[Ph_4_Pn] and Ph_4_PnH_2_, the phenyl substituents in Ph_4_Pn were twisted *versus* the plane of the Pn core by 31.7(3)–42.3(3) Å.^57,59^ This value was larger than in Mg[Ph_4_Pn] but marginally less than the non-aromatic Ph_4_PnH_2_ (Table 1). In Mg[Ph_4_Pn] the phenyl groups strive to adopt a co-planar arrangement to delocalise the negative charge as much as sterically possible,^67^ whilst in Ph_4_Pn and Ph_4_PnH_2_ there is no charge to distribute and so the phenyl groups likely relax in a slightly larger twist angle to minimise steric clash.

**Fig. 4:**
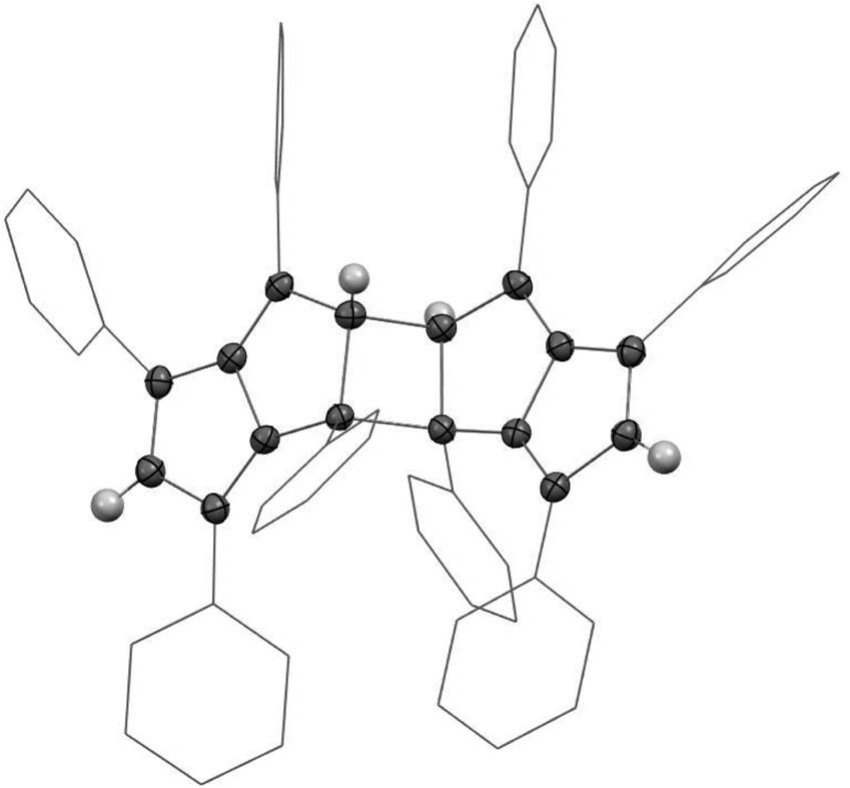
X-ray crystal structure of [1,3,4,6-Ph_4_Pn]_2_ with thermal ellipsoids at the 50% probability level (phenyl hydrogen atoms omitted for clarity).

Serendipitously, the [2 + 2] cycloaddition dimer [Ph_4_Pn]_2_ was found to co-crystallise with Ph_4_Pn in the same unit cell. In this structure two Pn units were connected *via* a cyclobutane bridge in an extended chair-like conformation (Fig. 4). The [2 + 2] cycloaddition means that [Ph_4_Pn]_2_ is formally non-aromatic with each 6π pentafulvene units containing two adjacent sp^3^ centres. The C=C bond lengths in [Ph_4_Pn]_2_ ranged between 1.357(2)–1.374(2) Å and are contracted relative to the anti-aromatic Ph_4_Pn (Table 1), more reminiscent of those found for the non-aromatic Ph_4_PnH_2_ which [Ph_4_Pn]_2_ is most closely related to.^57^ The sp^3^ bonds within the cyclobutane unit were significantly longer than the other sp^2^ bonds in the dimer, with bond lengths of 1.567(2)–1.675(2) Å. Sun and co-workers reported similar, but marginally shorter, bond lengths of the cyclobutane linkage (1.56–1.60 Å) for their [^c^Pr_4_Pn]_2_.^53^ O'Hare and co-workers reported that reaction of *cis*-(Me_3_Sn)_2_Pn* with FeCl_2_ resulted in oxidation of the pentalenide followed by dimerisation, however, no crystallographic data are available for [Pn*]_2_.^52^

Geometry optimisation of Ph_4_Pn at the B3LYP/6-311++g(d,p) level of theory in the gas phase gave good agreement with the crystallographic data, including the 39.2–44.5° twist angle of the phenyl groups. This suggests the origin of the rotation not to be due to packing effects in the solid state, but to arise so as to avoid overlap of the aromatic (phenyl groups) and antiaromatic (pentalene core) ring currents. The calculated C–C perimeter range of 1.37–1.48 Å for the pentalene core and 1.45 Å for the C–C bridge also matched well with the crystallographic data.

**Fig. 5:**
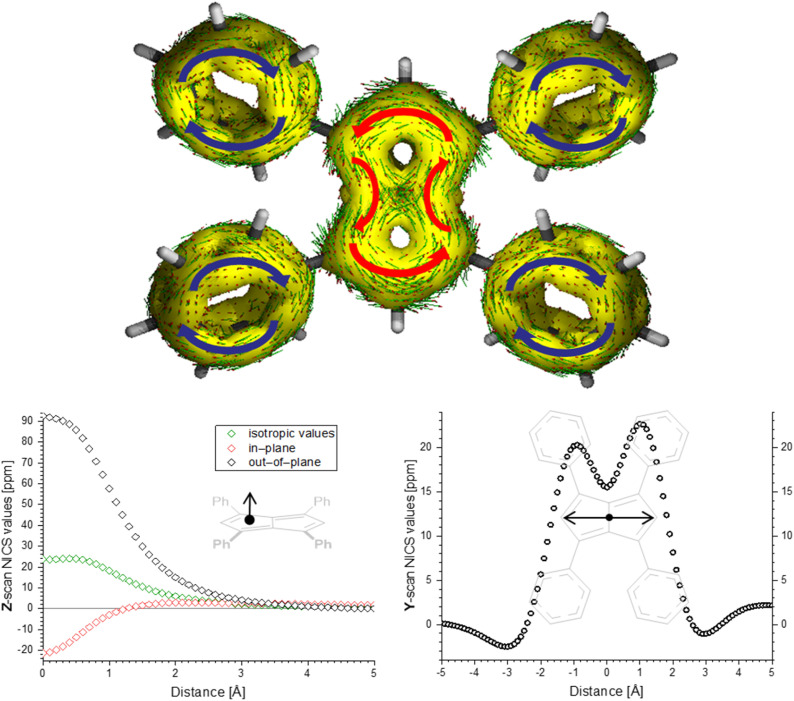
ACID plot ((top); iso-value = 0.03) and NICS scans ((bottom); *Z* scan [left] and *Y* scan [right], at a *Z* height of 1.7 Å; NICS probe BQ shown as •) of Ph_4_Pn.

To investigate the extent of antiaromaticity in Ph_4_Pn, harmonic oscillator model of aromaticity (HOMA) calculations were performed using the experimental X-ray data, alongside DFT calculations of the anisotropy of the induced current density (ACID) and calculations on the nucleus independent chemical shift (NICS) along different axes of the molecule (Fig. 5). A HOMA_B_ value of 0.28 was found for the pentalene core, with the phenyl groups returning a value of 0.99 indicating no overlap of the two π-systems. The lack of overlap is also reflected in the NICS scan along the *Z*-axis through the centre of one phenyl group. This returned a value for the out-of-plane component of the chemical shift of −26.34 ppm at *Z* = 0.7 Å, which is very close to that of unsubstituted benzene (Fig. S6†).^68^ In contrast to the dianionic tetraphenylpentalenide,^67^ the ESP map of neutral Ph_4_Pn showed an equally distributed charge density over the pentalene core and the flanking phenyl groups (Fig. S10†). The aromatic pentalenide core of Ph_4_Pn^2−^ gave a HOMA_B_ value of 0.63, showing a clear change in aromaticity between the 8π and 10π versions of the molecule. The ACID plot of Ph_4_Pn showed a strong paratropic ring current about the perimeter of the pentalene core with a significant contribution from the transannular C–C bridge. The diatropic ring currents of the four aromatic phenyl substituents were clearly separated and essentially unperturbed by the presence of the anti-aromatic core, as indicated by the corresponding HOMA_B_ values. The NICS scan along the *Z*-axis, starting from the centre of one 5-membered ring running perpendicular to the pentalene plane, showed features characteristic of an antiaromatic π system (Fig. 5). The isotropic shift values were positive throughout the scan, with a maximum of 24.15 ppm at *Z* = 0.4 Å, and the shape of the curve was strongly dominated by the out-of-plane component of the chemical shift. The NICS-*Y* scan, running parallel to the pentalene plane at a height of 1.7 Å revealed two maxima at *Y* = −0.9 Å (20.28 ppm) and at *Y* = 1 Å (22.67 ppm) close to the centres of each 5-membered ring, consistent with the paratropic current found in the ACID plot, and mirrors that of the strongly aromatic dianion Ph_4_Pn^2−^.^67^ These maxima were separated by a local minimum at the centre of the molecule around the transannular C–C bridge (*Y* = 0 Å, 15.50 ppm) as a result of two contributions: a paratropic current about the perimeter of the pentalene system combined with two local paratropic currents in each 5-membered ring. As observed by Stanger *et al.* for unsubstituted pentalene, the latter contribution typically dominates in these systems,^69^ although retaining some global antiaromaticity. This strong influence of local currents in the two 5-membered rings can also be seen by the turbulence around the transannular C–C bridge in the ACID plot of Ph_4_Pn (Fig. 5). The NICS and ACID calculations for the [2 + 2] cycloaddition product [Ph_4_Pn]_2_ showed a complete loss of the antiaromatic current (Fig. S12;† with only the flanking phenyl rings retaining their aromatic character), consistent with the notion that the dimerisation of pentalenes is driven by a relief of antiaromatic destabilisation.

**Fig. 6:**
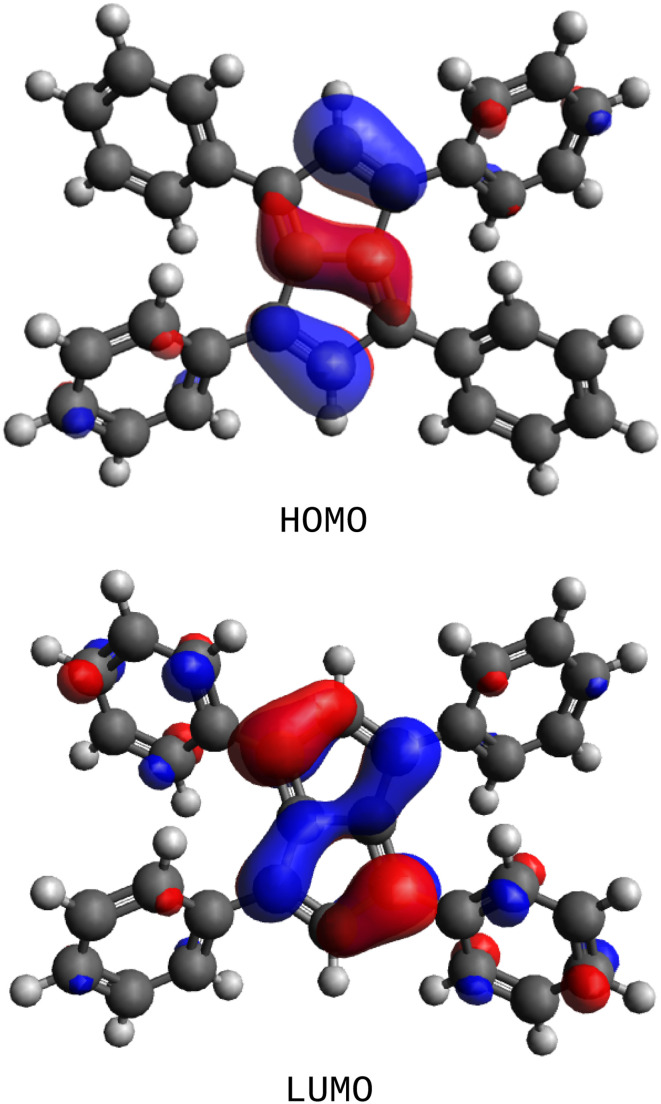
Frontier molecular orbitals of Ph_4_Pn.

The computed frontier orbitals of Ph_4_Pn (Fig. 6) agree well with those calculated for unsubstituted Pn.^70^ Both the HOMO and the LUMO are characterised as π orbitals of the pentalene system, with minimal contribution from the substituents. The HOMO showed major contributions from the localised trisubstituted C=C bond in each C_5_-ring, polarised towards the wingtip carbon, as well as a noticeable contribution from the transannular C–C bond. In contrast, in the HOMO of Ph_4_Pn^2−^ the largest contributions were found to be localised on the 1,3,4,6-positions.^67^ The LUMO of Ph_4_Pn showed increased delocalisation across the transannular bond into the 1,4-*ipso*-carbons but (unlike in Ph_4_Pn^2−^) without any delocalisation into the phenyl substituents. The HOMO–LUMO gap for Ph_4_Pn of 2.11 eV was about 1 eV smaller than that of Ph_4_Pn^2−^ (3.15 eV), indicating a more reactive system due to the antiaromatic core. Pn has been calculated to have a HOMO–LUMO gap of 1.12 eV,^70^ 1 eV smaller than for Ph_4_Pn emphasising the stabilising influence of the four phenyl substituents. A singlet-triplet gap of 41.1 kJ mol^−1^ (0.43 eV) was calculated for Ph_4_Pn, about four times greater than that computed for Pn (0.1 eV),^71^ highlighting the decreased propensity of Ph_4_Pn to dimerise through increased kinetic stabilisation of the singlet state.

**Fig. 7:**
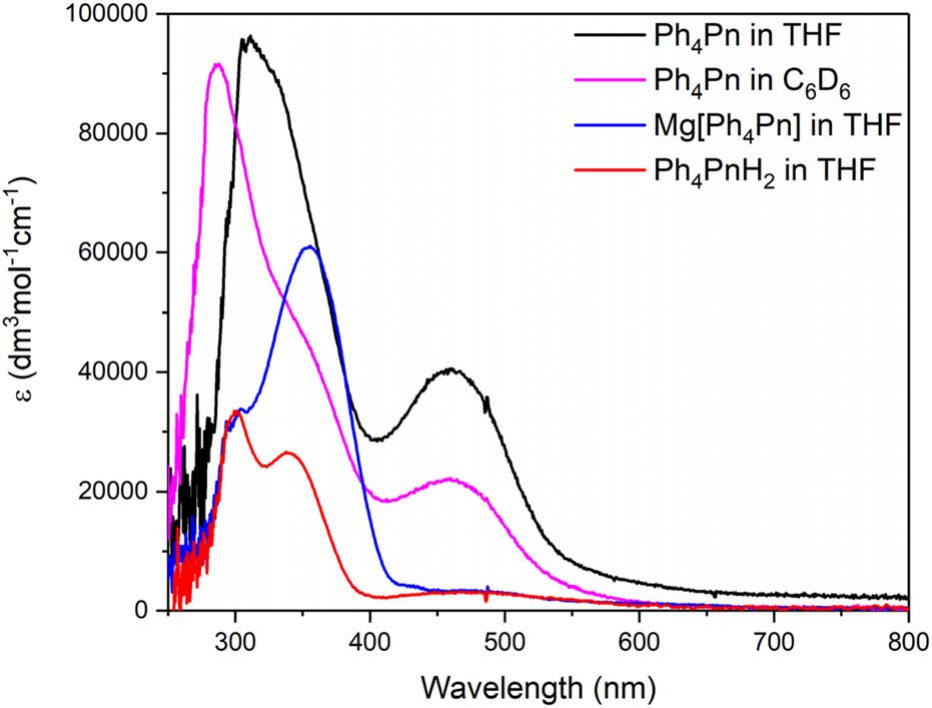
UV-vis spectra of 1,3,4,6-Ph_4_Pn (6 μM), Mg[Ph_4_Pn] (15 μM) and Ph_4_PnH_2_ (11 μM) in THF at 298 K.

The UV-vis spectrum of Ph_4_Pn in THF gave rise to two broad absorptions (Fig. 7). The first band at *λ*_max_ = 312 nm (*ε* = 95 000 M^−1^ cm^−1^) was nearly three times more intense than comparable absorptions observed for Ph_4_PnH_2_ (*λ* = 301, *ε* = 31 500 M^−1^ cm^−1^ and *λ* = 339 nm, *ε* = 26 800 M^−1^ cm^−1^) and are attributed to electronic transitions from the HOMO−1 into the LUMO+2 and LUMO+3 on the basis of TD-DFT (Fig. S16†). The second maximum at *λ*_max_ = 460 nm (*ε* = 40 000 M^−1^ cm^−1^) is assigned as a HOMO−1 → LUMO transition. A similar absorption was seen for Ph_4_PnH_2_ at 468 nm, though an order of magnitude less intense (*ε* = 3600 M^−1^ cm^−1^), and was assigned to the fulvene-like double bond in the molecule.^57^ The UV-vis spectrum of Ph_4_Pn in benzene largely retained the same features as in THF, indicating there is no significant change in the solution structure of Ph_4_Pn between the two solvents, consistent with the NMR data. The main band at *λ*_max_ = 275 nm was of slightly lower intensity (*ε* = 90 000 M^−1^ cm^−1^) and blue shifted by 50 nm compared to the same absorption in THF. A marginally more pronounced shoulder at *λ*_max_ = 350 nm was seen in the benzene spectrum and accounted for by TD-DFT (Fig. S16†). Dilute solutions of Ph_4_Pn in THF and benzene were both pale yellow due to the absorption at 460 nm, even though in benzene this band was only about half the intensity than in THF. 1,3,5-*^t^*Bu_3_Pn has been reported to be dark blue in colour with a visible absorption at 598 nm, and 1,3-(NMe_2_)_2_Pn, 1,4-Me_2_-2,5-(CN)_2_-3,6-(NH_2_)_2_Pn and Ph_6_Pn were also reported to have absorptions >600 nm. Similar low energy absorptions were found computationally for Ph_4_Pn but with much lower intensities (Fig. S16 and S17†).

**Fig. 8:**
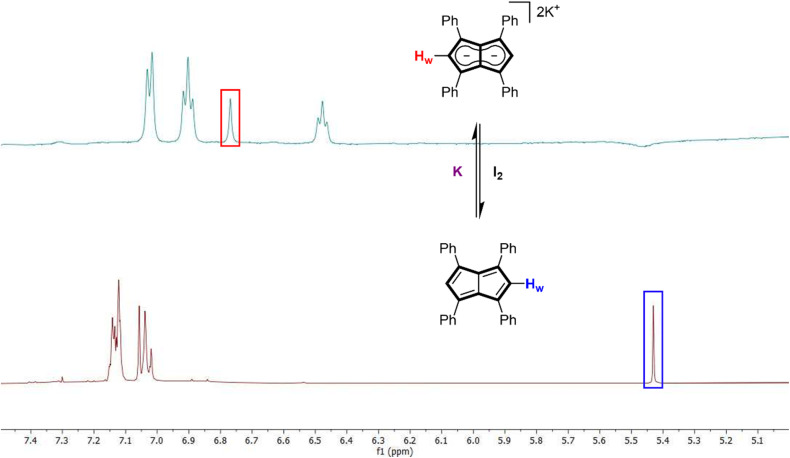
500 MHz ^1^H NMR spectra of K_2_[Ph_4_Pn] (top) and 1,3,4,6-Ph_4_Pn (bottom).

In line with the electrochemistry of Ph_4_Pn showing a reduction peak ∼0.3 V *vs.* NHE in THF (Fig. S18†), Ph_4_Pn can be reduced back to Ph_4_Pn^2−^ by contacting a THF solution of Ph_4_Pn with a freshly prepared potassium mirror, causing an immediate colour change from dark yellow to bright red. The ^1^H NMR spectrum of the solution showed full consumption of Ph_4_Pn and the spectroscopically quantitative formation of K_2_[Ph_4_Pn] (Fig. 8) with a characteristic H_w_ shift of 6.76 ppm.^57^ The ease of the reduction back to the aromatic Ph_4_Pn^2−^ is supported by DFT where the enthalpy of Ph_4_Pn was found to be 72.8 kJ mol^−1^ higher than Ph_4_Pn^2−^.

## 3. Conclusion

A stable pentalene has been synthesised through the facile oxidation of its corresponding pentalenide using elemental iodine. Unlike previous reports of pentalenes, Ph_4_Pn crystallised as a mixture of both the monomer and dimer in the same unit cell. However, NMR spectroscopy showed that in solution the monomeric form dominates, and UV-vis spectroscopy indicated no solvatochromism or change in the solution structure in THF and benzene. Ph_4_Pn is a rare example of a small molecule featuring both Hückel aromatic and antiaromatic subunits in close proximity, albeit electronically separated from each other as shown by quantum chemical calculations. Therefore, the stabilisation of the anti-aromatic pentalene core imparted by the four aromatic phenyl substituents is largely steric in nature. The reduction of Ph_4_Pn back to Ph_4_Pn^2−^ was found to be reversible within a potential range of ∼1 V, representing a unique example of a small hydrocarbon that can easily switch between an aromatic and anti-aromatic state without any conformational or skeletal rearrangements. These properties alongside its structural simplicity and ease of synthesis^58,67^ suggest that Ph_4_Pn and its derivatives could find applications similar to those of dibenzopentalenes in electrochemical sensing or optoelectronics. In the context of organometallic chemistry, the facile oxidation of Ph_4_Pn^2−^ by iodine provides a useful threshold for the redox potential of metal ions bound to it to guide the design and synthesis of novel pentalenide complexes.

## Data availability

Crystallographic datasets are available from the CCDC deposition number 2335451, and other analytical data can be found in the ESI.†

## Author contributions

HJS carried out all synthetic work, spectroscopic analyses, electrochemistry, and drafted the manuscript. GKK conducted all XRD analyses. AH carried out all calculations with guidance and supervision by HH. UH led the project, and all authors contributed to refining the manuscript.

## Conflicts of interest

The authors declare no conflicts of interest.

## Supplementary Material

SC-OLF-D4SC06439A-s001

SC-OLF-D4SC06439A-s002

SC-OLF-D4SC06439A-s003

SC-OLF-D4SC06439A-s004

SC-OLF-D4SC06439A-s005

SC-OLF-D4SC06439A-s006
