# Supplementary material for: Reversible formation of tetraphenylpentalene, a room temperature stable antiaromatic hydrocarbon
Source: Chem Sci. 2024 Nov 25;16(2):952–61. doi: 10.1039/d4sc06439a (PMC11627289; doi:10.1039/d4sc06439a)
Supplement: SC-016-D4SC06439A-s004 [file SC-016-D4SC06439A-s004.pdf]

Supplementary Information (SI) for Chemical Science.
This journal is © The Royal Society of Chemistry 2024

# Reversible formation of tetraphenylpentalene, a room temperature stable antiaromatic hydrocarbon

Hugh J. Sanderson,[a] Andreas Helbig,[b] Gabriele Kociok-Köhn,[c] Holger Helten,*[b] Ulrich Hintermair*[a]

a) *Department of Chemistry and Institute for Sustainability, University of Bath, Claverton Down, Bath BA2 7AY, UK.*
b) *Julius-Maximilians-Universität Würzburg, Institute of Inorganic Chemistry and Institute for Sustainable Chemistry & Catalysis with Boron, Am Hubland, 97074 Würzburg, Germany*
c) *Physical Structure Characterisation, University of Bath, Claverton Down, Bath BA2 7AY, UK.*

*holger.helten@uni-wuerzburg.de
*u.hintermair@bath.ac.uk

# Supporting Information

## Contents

Experimental ..... 2
Spectroscopic Data ..... 4
  1,3,4,6-tetraphenylpentalene ..... 4
  Reduction with Potassium ..... 6
Computational ..... 6
Electrochemistry ..... 15
Crystallographic Data ..... 18
References ..... 19

# Experimental

**General Considerations:** All reactions were conducted under argon using standard Schlenk techniques or a MBraun Unilab Plus glovebox, unless stated otherwise. All commercially available materials were purchased from Sigma Aldrich, Fisher or Acros.

**Solvents:** Methanol was dried and distilled over magnesium. Toluene was dried and distilled over sodium. THF, hexane and pentane were dried and distilled over potassium. $C_6D_6$ was distilled from $CaH_2$ and stored over 4Å molecular sieves.

**Reagents:** 1,3,4,6-tetraphenyl-1,2-dihydropentalene[1] and magnesium 1,3,4,6-tetraphenylpentalenide[2] were prepared according to literature procedures.

**Analysis:** NMR spectra were obtained using either a 500 MHz Bruker Avance III or a 400 MHz Bruker AvanceNeo spectrometer. Chemical shifts (δ) are given in ppm and referenced to residual proton chemical shifts from the NMR solvent for $^{1}H$ and $^{13}C\{^{1}H\}$ spectra. IR spectra were recorded on a Bruker Alpha II FT-IR spectrometer using a diamond ATR module. UV-vis spectroscopy was performed inside a MBraun Unilab Plus glovebox using an Avantes AvaLight-DH-S-BAL light source and an AvaSpec-2048L photospectrometer. Data was collected between 250 nm and 1000 nm with an integration time of 4 ms.

Single crystal X-ray diffraction analysis was carried out using a RIGAKU SuperNova, Dual,Cu a zero EoS2 single crystal diffractometer. Mass spectrometry was carried out at the Chemical Characterisation Facility at the University of Bath using a Bruker MaXis HD ESI-QTOF.

**Computational methods:** Optimization, NICS Scan[3], NICS XY-Scan,[4] ACID,[5,6] and TD-DFT calculations were carried out using the Gaussian 09 program package, revision D.01.[7] Becke's three parameter exchange-correlation hybrid functional B3LYP[8–12] was used in combination with the 6-311++G(d,p) basis set. The stationary points were characterized as minima by an analytical vibrational frequency calculation[13,14] which revealed the absence of imaginary frequencies. The NICS Z scan was performed perpendicular to the respective ring, from its center up to 4 Å above the plane, and the NICS Y scan at 1.7 Å above the respective subunits. The ACID calculation was plotted with an isovalue of 0.03. The optimised structure as well as the calculation setup for the NICS Z and the NICS Y scan are attached as .mol-files.

***Synthesis of 1,3,4,6-tetraphenylpentalene***

A freshly prepared THF solution of iodine (0.1 M, 1.5 mL, 0.15 mmol) was added to a THF solution of **Mg[$Ph_4$Pn]** (98 mg, 0.15 mmol) at room temperature. An immediate colour change from orange to dark red was observed followed by precipitation of a yellow solid. The reaction was allowed to stir for one hour before it was filtered, and the solution evaporated to dryness and kept under vacuum overnight to yield **1,3,4,6-$Ph_4$Pn** as a dark red powder (46 mg, 75%)

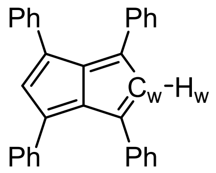


**$^1$H NMR** (500 MHz, THF-$h_8$) δ: 7.15-7.13 (m, 12H, ArH), 7.07-7.04 (m, 8H, ArH), 5.44 (s, 2H, $H_w$)

**$^{13}$C{$^1$H} NMR** (126 MHz, THF-$h_8$) δ: 147.9, 145.6, 135.1, 134.9 ($C_w$), 129.2, 128.5, 127.6

**HRMS (APCI) *m/z*:** $[M+H]^+$ Calculated for $C_{32}H_{23}$: 407.1794, Found: 407.1767

**UV-vis (THF)** λ: 312 nm (ε = 95,000 $M^{-1}cm^{-1}$), 460 nm (ε = 40,000 $M^{-1}cm^{-1}$)

**UV-vis ($C_6D_6$)** λ: 275 nm (ε = 90,000 $M^{-1}cm^{-1}$), 460 nm (ε = 20,000 $M^{-1}cm^{-1}$)

## Spectroscopic Data

### 1,3,4,6-tetraphenylpentalene

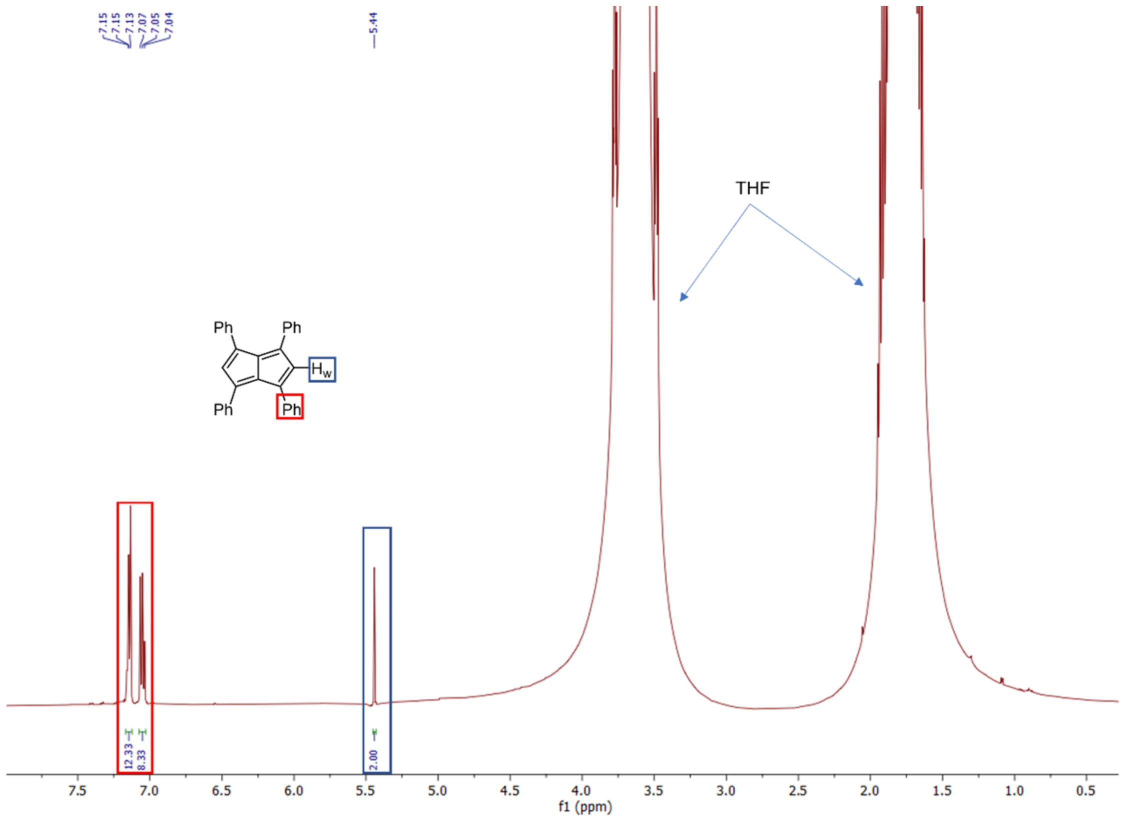


**Figure S1:** 500 MHz $^{1}H$ NMR spectrum of 1,3,4,6-tetraphenylpentalene in THF-$H_8$ recorded at room temperature.

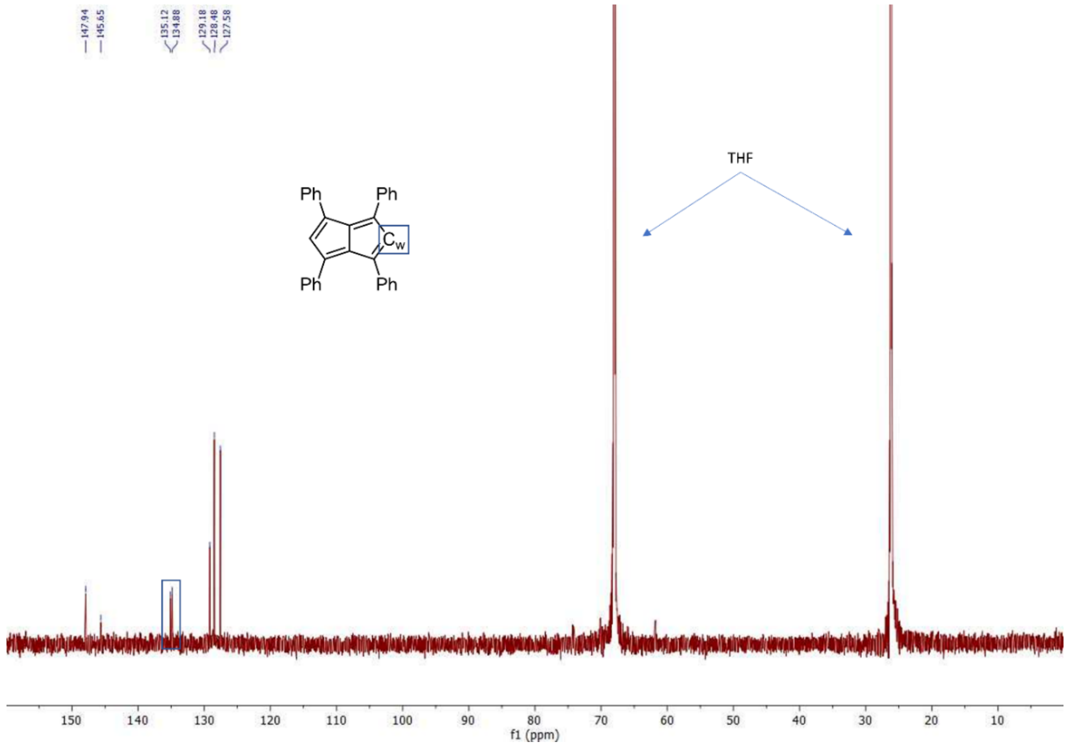


**Figure S2:** 125 MHz $^{13}C\{^{1}H\}$ NMR spectrum of **$Ph_4Pn$** in THF-$H_8$ recorded at room temperature.

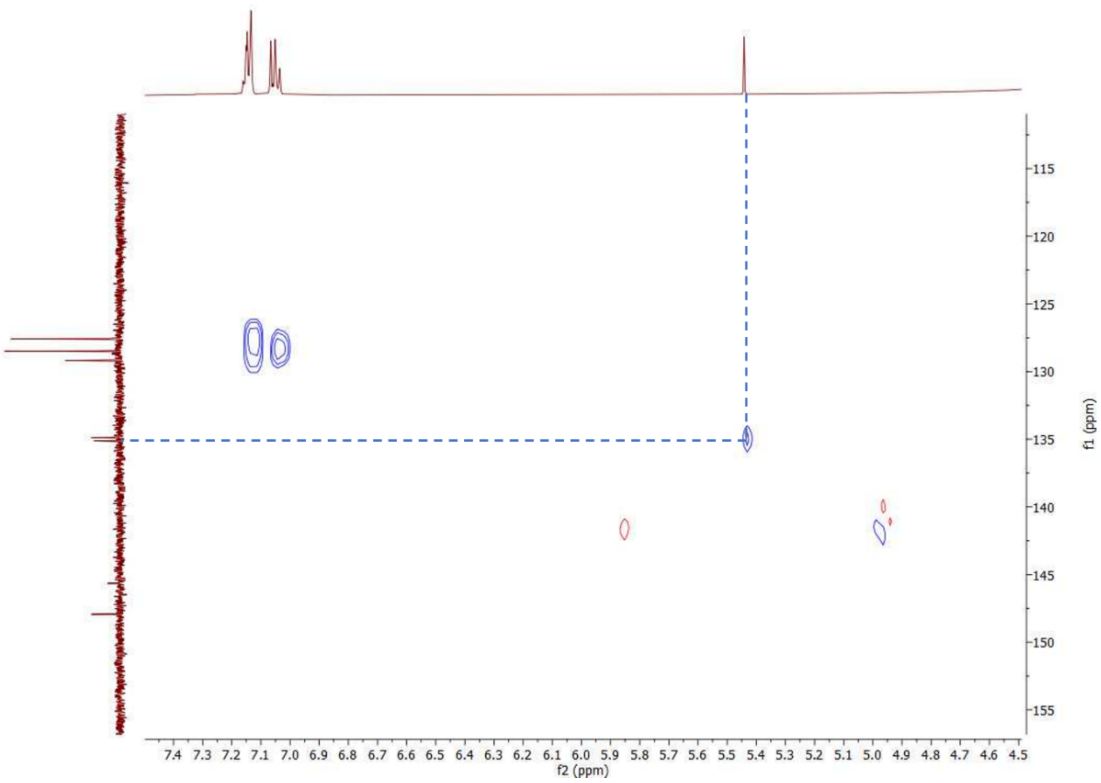


**Figure S3:** 500 MHz $^1H$-$^{13}C$ HSQC spectrum of 1,3,4,6-tetraphenylpentalene in THF-$H_8$ ($H_w$ = blue)

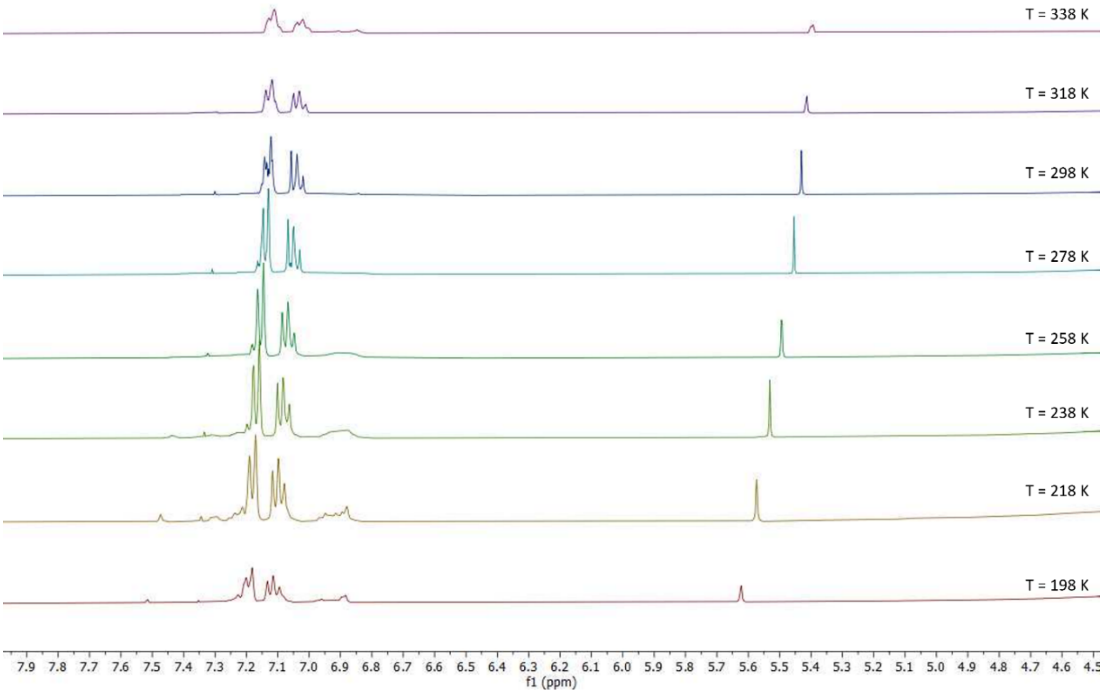


**Figure S4:** 400 MHz $^1H$ variable temperature NMR spectra of 1,3,4,6-tetraphenylpentalene in THF-$H_8$

Reduction with Potassium

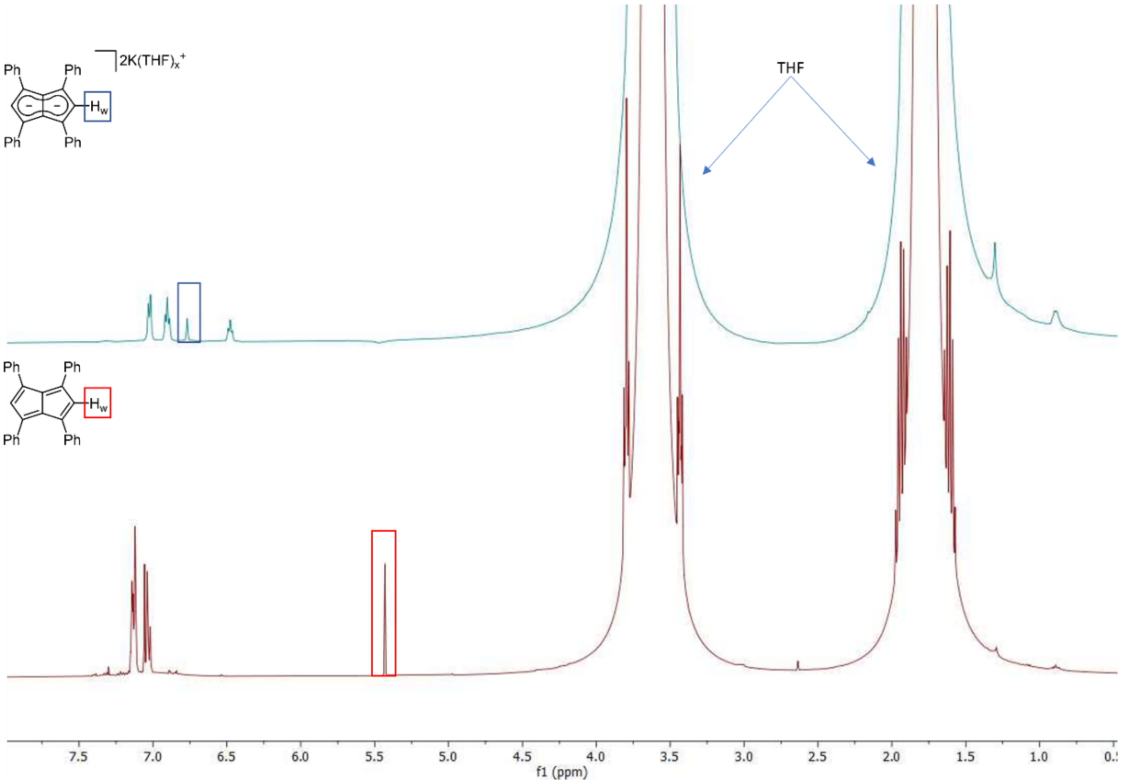


**Figure S5:** 500 MHz $^1$H NMR spectra of **$K_2[Ph_4Pn]$** (top) formed by reaction of **$Ph_4Pn$** (bottom) with potassium in THF at room temperature.

# Computational

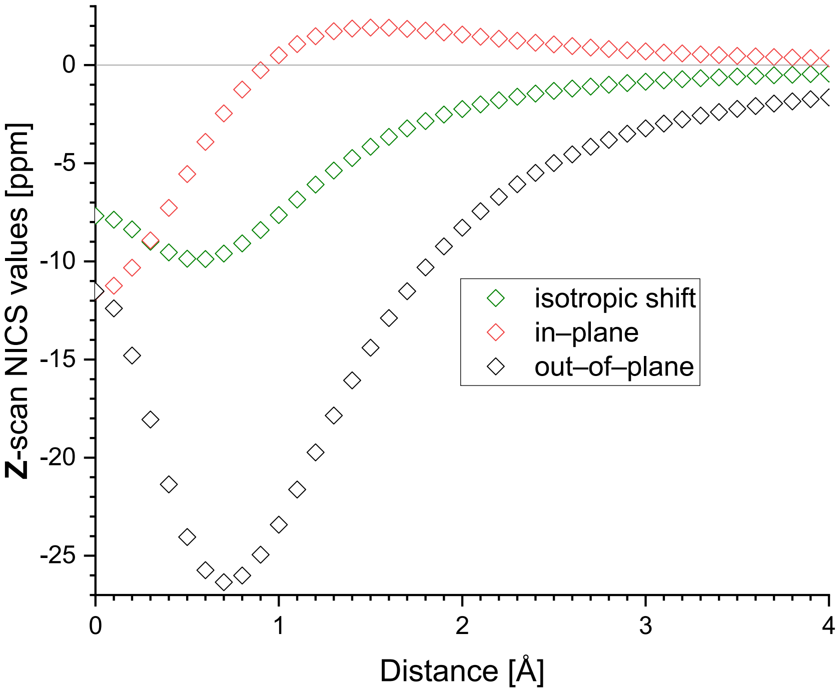


**Figure S6:** NICS Z scan through the centre of one of the flanking phenyl rings of **$Ph_4Pn$** using DFT calculations at the B3LYP/6-311++g(d,p) level of theory.

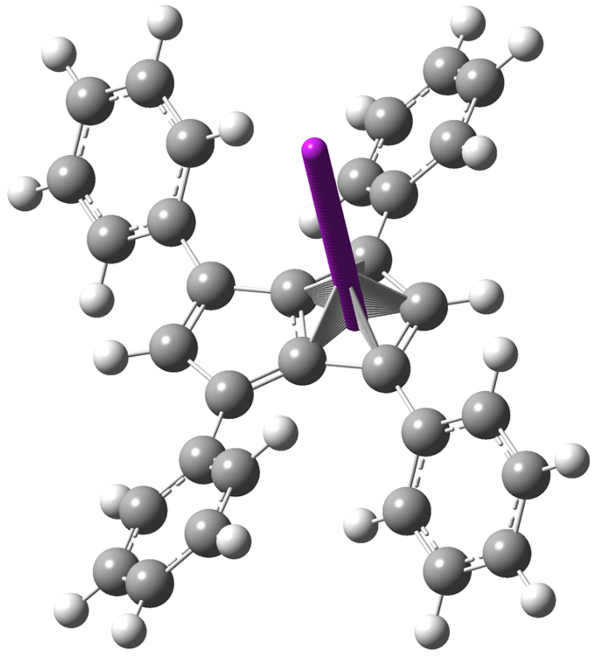

**Figure S7:** Placement of the Bq atoms for the NICS Z scan of the pentalene core of **$Ph_4Pn$**.

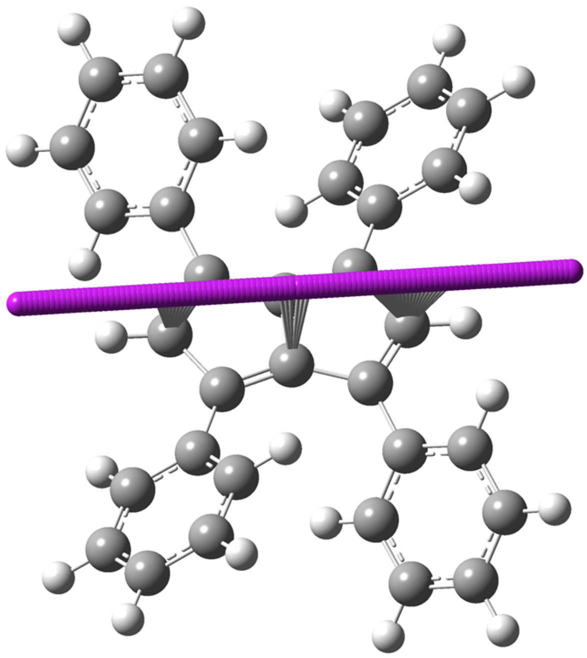

**Figure S8:** Placement of the Bq atoms for the NICS Y scan of the pentalene core of **$Ph_4Pn$**.

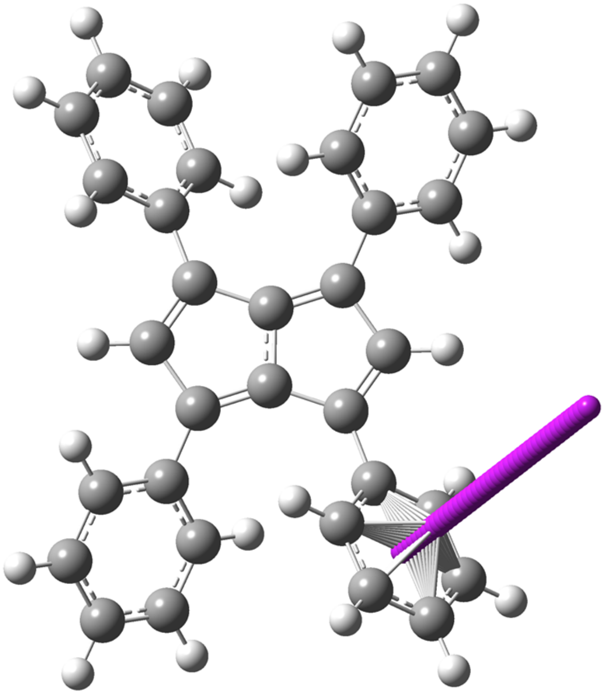

**Figure S9:** Placement of the Bq atoms for the NICS Z scan of one phenyl moiety of **$Ph_4Pn$**.

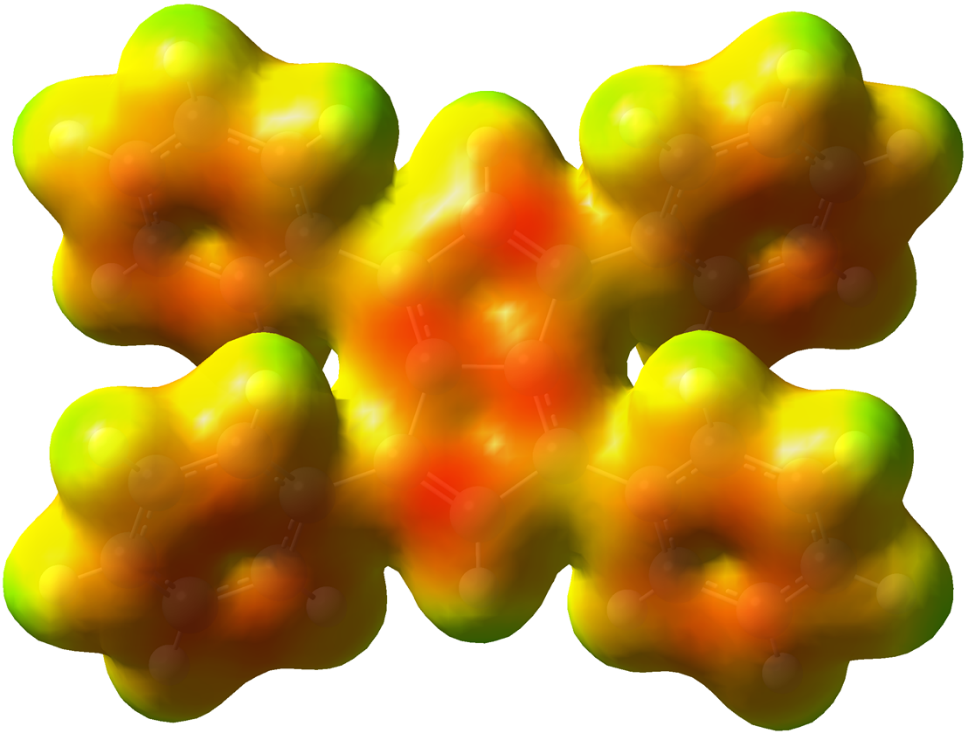

**Figure S10:** Electrostatic potential (ESP) map for **$Ph_4Pn$**. Contour colours range from −0.036 (red) to 0.3 (blue) at an isovalue of 0.01.

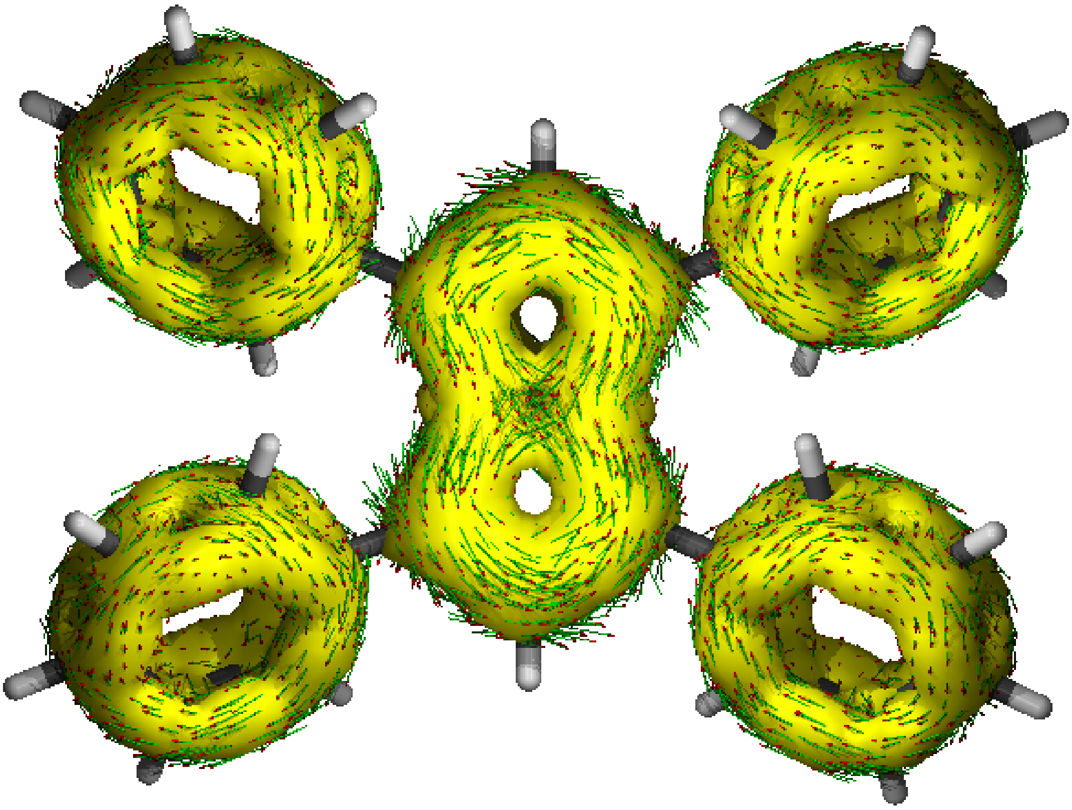

**Figure S11:** ACID plot of **$Ph_4Pn$** visualized with an isovalue of 0.03. Calculated using DFT calculations at the B3LYP/6-311++g(d,p) level of theory.

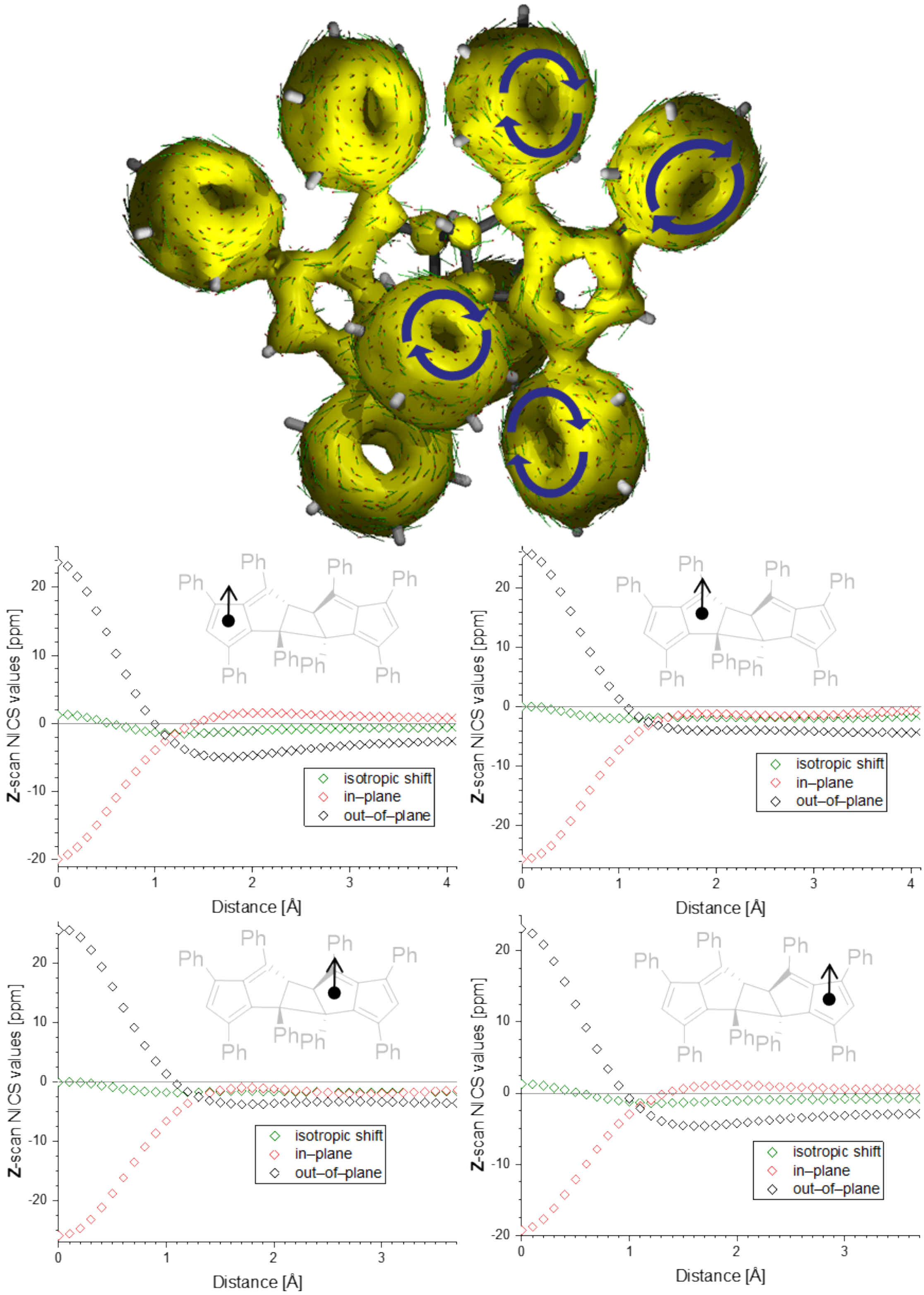


**Figure S12:** ACID plot (top; iso-value = 0.015) and NICS Z scans of the respective five-membered cycle in **$[Ph_4Pn]_2$** indicated by the positioning of the arrow in the structure; NICS probe BQ shown as • of the cycloaddition product. All calculated using DFT calculations at the B3LYP/6-311++g(d,p) level of theory.

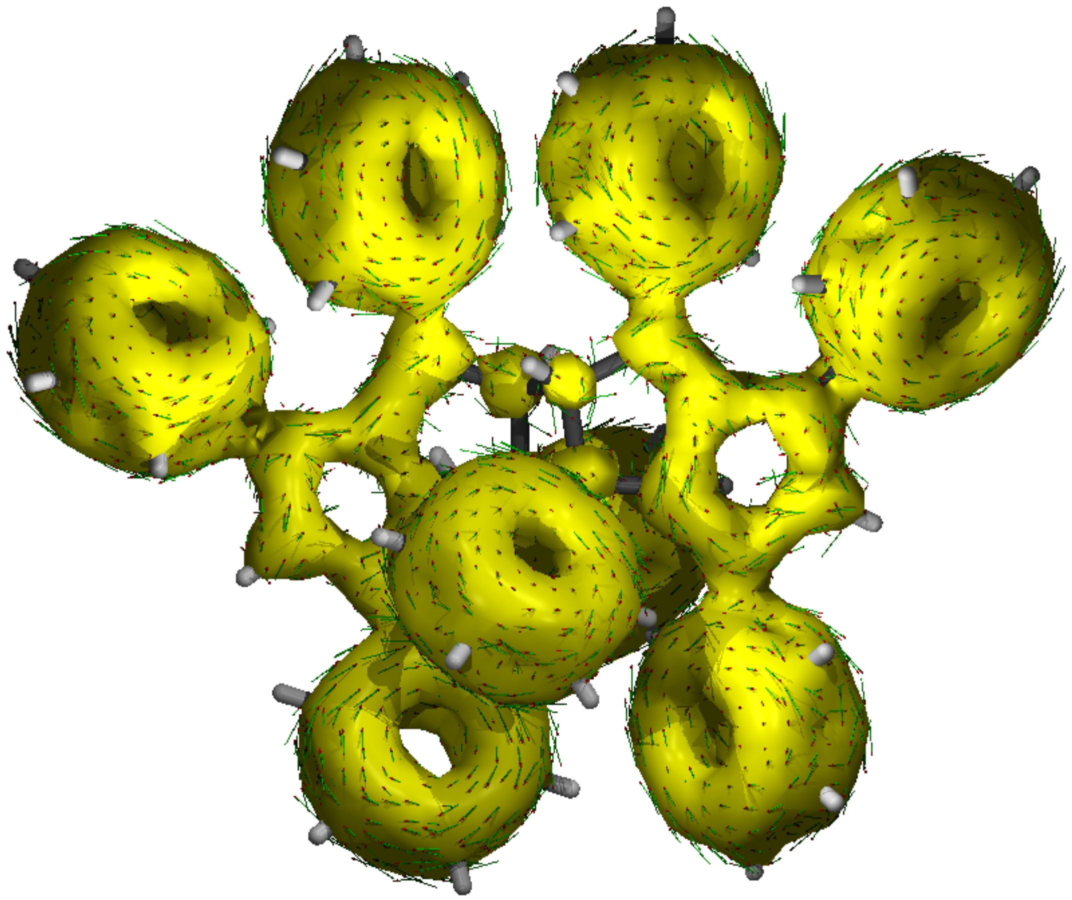

**Figure S13:** ACID plot of the cycloaddition product **[$Ph_4Pn$]$_2$** visualized with an isovalue of 0.015. Calculated using DFT calculations at the B3LYP/6-311++g(d,p) level of theory.

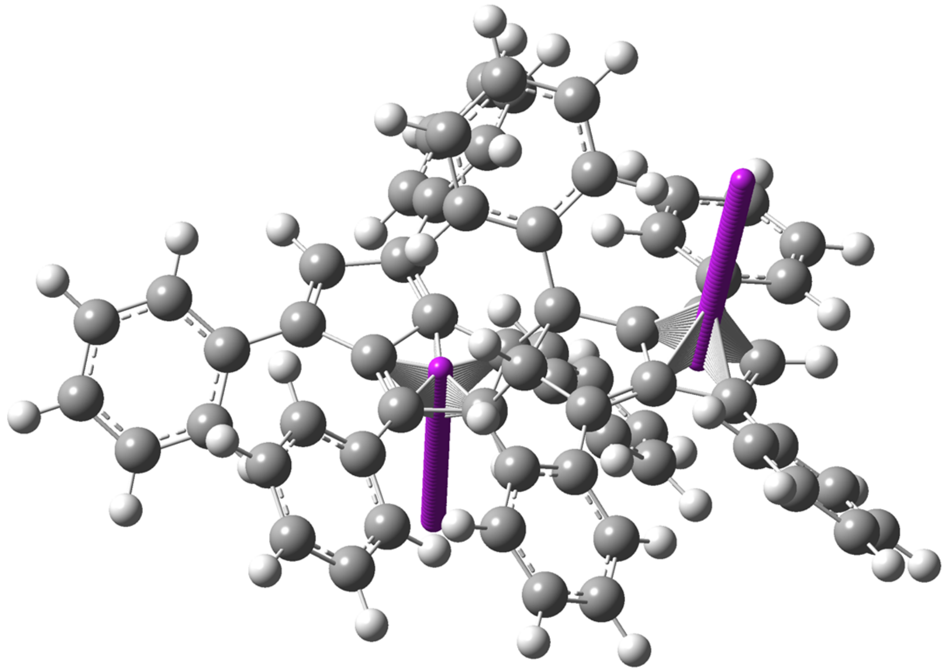

**Figure S14:** Placement of the Bq atoms for the NICS Z scan of two five-membered rings in **[$Ph_4Pn$]$_2$**.

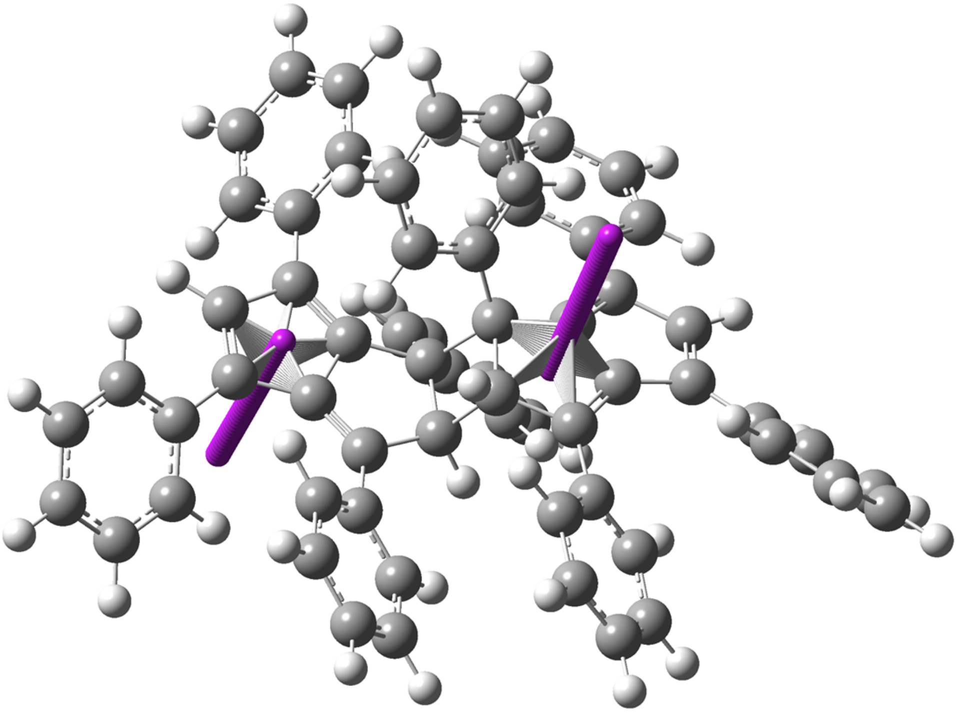

**Figure S15:** Placement of the Bq atoms for the NICS Z scan of the other two five-membered rings in **$[Ph_4Pn]_2$**.

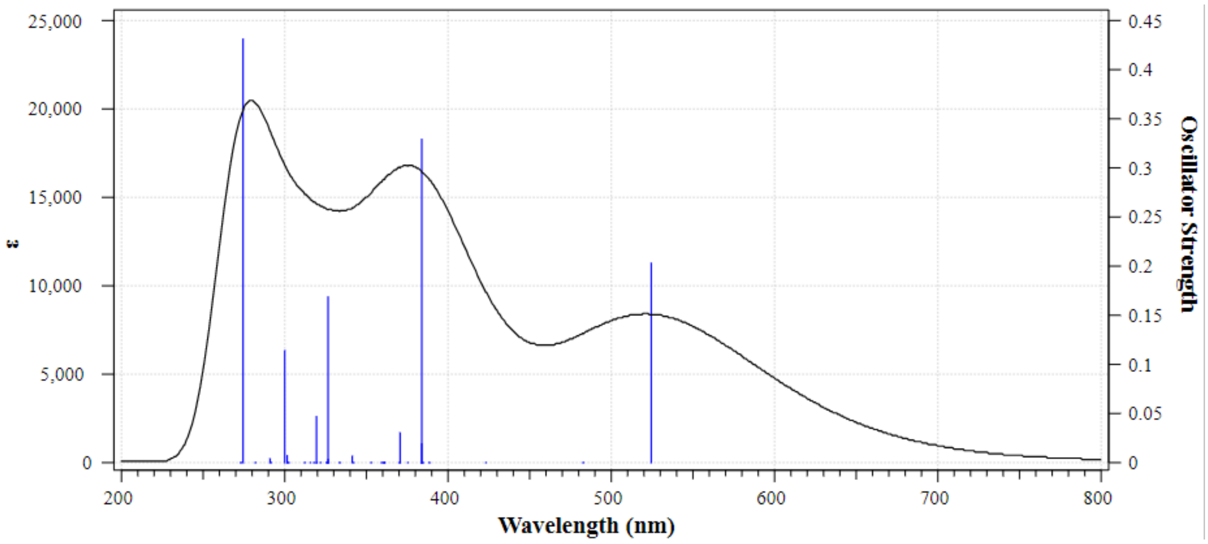


**Figure S16:** TD-DFT of singlet **$Ph_4Pn$** performed at the B3LYP/6-311++g(d,p) level of theory in the gas phase. Graphic was obtained via the standard Gaussian plot function.

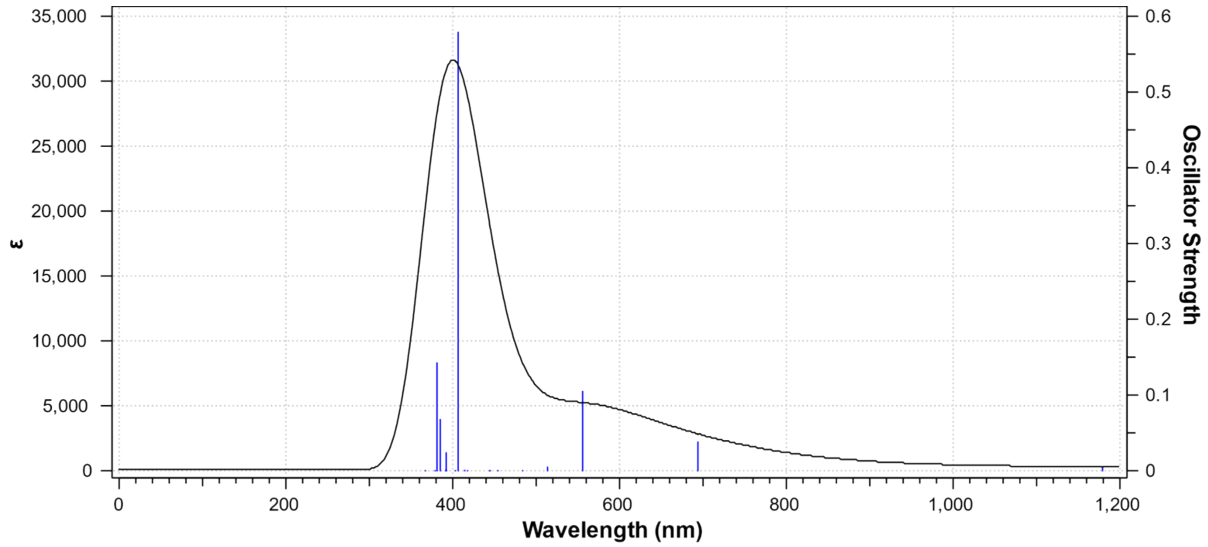


**Figure S17:** TD-DFT of triplet **$Ph_4Pn$** performed at the B3LYP/6-311++g(d,p) level of theory in the gas phase. Graphic was obtained via the standard Gaussian plot function.

# Electrochemistry

Cyclic voltammetry was performed inside a MBraun Unilab Plus Glovebox using a three-electrode cell and an Ivium Technologies CompactStat. 0.1 M $[NBu_4][PF_6]$ electrolyte in THF, a graphitic carbon working electrode, platinum wire counter electrode and a $Ag/Ag^+$ pseudo reference electrode (0.01 M $AgPF_6$ in THF) were used at ambient temperature. Potentials were referenced to the normal hydrogen electrode (NHE) by means of the following equation:[15]

$$E_{(NHE)} = E_{(Ag/AgNO_3)} + 0.197$$

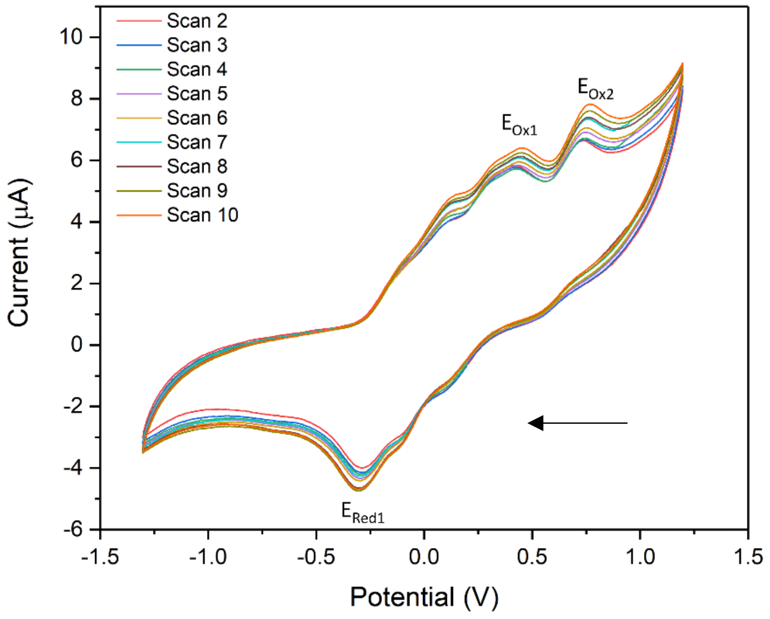


$E_{Red1} = Ph_4Pn + 2e^- \rightarrow Ph_4Pn^{2-}$

$E_{Ox1} = Ph_4Pn^{2-} \rightarrow Ph_4Pn^{\cdot -} + e^-$

$E_{Ox2} = Ph_4Pn^{\cdot -} \rightarrow Ph_4Pn + e^-$

**Figure S18:** CV of **$Ph_4Pn$** in THF at 1.6 mM recorded at a scan rate of 25 $mVs^{-1}$ with a step size of 5 mV.

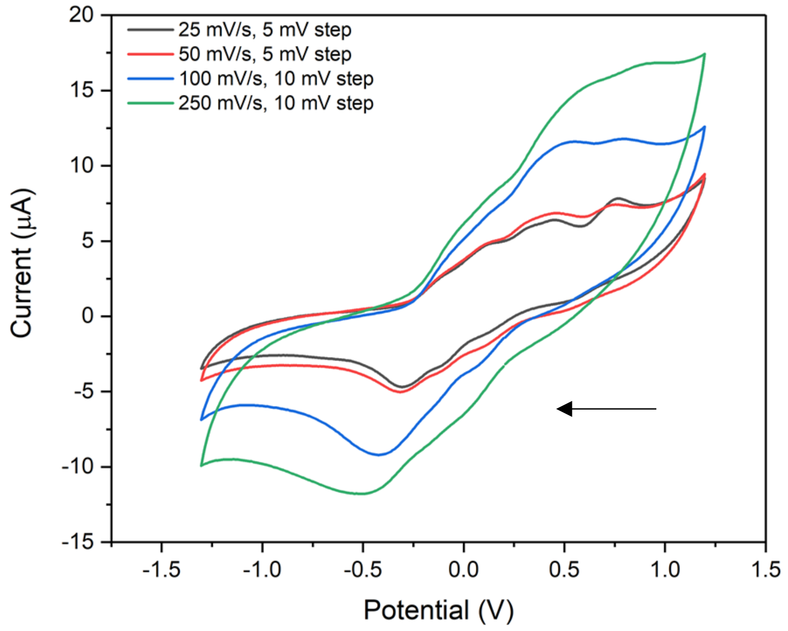


**Figure S19:** CV of **$Ph_4Pn$** recorded at varying scan rates.

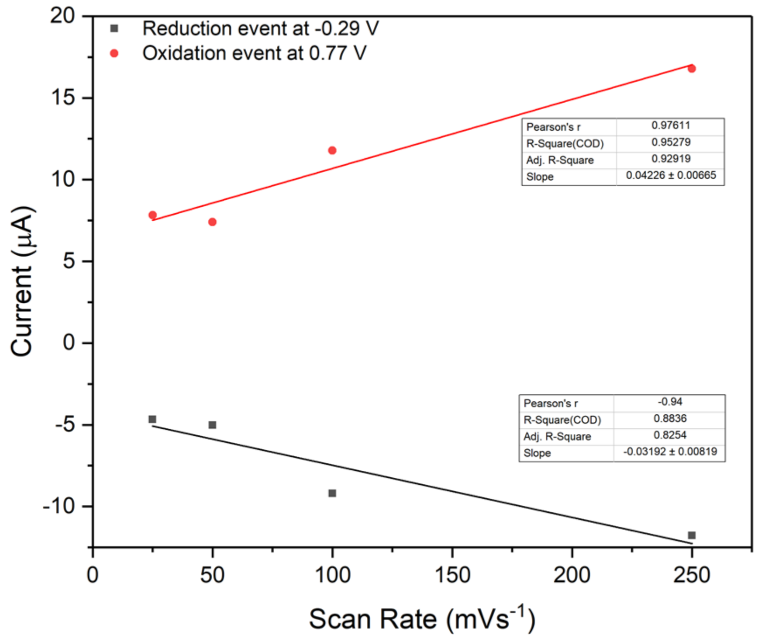


**Figure S20:** Randles-Sevcik plot of **$Ph_4Pn$**.

# Crystallographic Data

| | | |
|---|---|---|
| **CCDC** | **2335451** | |
| Identification code | s21uh10 | |
| Empirical formula | $C_{166} H_{123}$ | |
| Formula weight | 2117.64 | |
| Temperature | 150.00(10) K | |
| Wavelength | 1.54184 Å | |
| Crystal system | monoclinic | |
| Space group | C2/c | |
| Unit cell dimensions | a = 43.6143(9) Å | α = 90° |
| | b = 10.5720(2) Å | β = 98.309(2)° |
| | c = 25.1335(7) Å | γ = 90° |
| Volume | 11467.2(5) $Å^3$ | |
| Z | 4 | |
| Density (calculated) | 1.227 $Mg/m^3$ | |
| Absorption coefficient | 0.523 $mm^{-1}$ | |
| F(000) | 4476 | |
| Crystal size | 0.279 x 0.110 x 0.020 $mm^3$ | |
| Theta range for data collection | 4.306 to 73.064° | |
| Index ranges | -53<=h<=51, -8<=k<=13, -30<=l<=31 | |
| Reflections collected | 49934 | |
| Independent reflections | 11373 [R(int) = 0.0570] | |
| Completeness to theta = 67.684° | 100.0 % | |
| Absorption correction | Semi-empirical from equivalents | |
| Max. and min. transmission | 1.00000 and 0.89168 | |
| Refinement method | Full-matrix least-squares on $F^2$ | |
| Data / restraints / parameters | 11373 / 36 / 778 | |
| Goodness-of-fit on $F^2$ | 1.012 | |
| Final R indices [I>2sigma(I)] | R1 = 0.0449, wR2 = 0.0994 | |
| R indices (all data) | R1 = 0.0655, wR2 = 0.1093 | |
| Extinction coefficient | n/a | |
| Largest diff. peak and hole | 0.216 and -0.223 e.$Å^{-3}$ | |

# References


(1) Boyt, S. M.; Jenek, N. A.; Sanderson, H. J.; Kociok-Köhn, G.; Hintermair, U. Synthesis of a Tetraphenyl-Substituted Dihydropentalene and Its Alkali Metal Hydropentalenide and Pentalenide Complexes. *Organometallics* **2022**, *41* (3), 211–225. https://doi.org/10.1021/acs.organomet.1c00495.

(2) Sanderson, H. J.; Kociok-Köhn, G.; Hintermair, U. Synthesis, Structure, and Reactivity of Magnesium Pentalenides. *Inorg. Chem.* **2023**, *62* (39), 15983–15991. https://doi.org/10.1021/ACS.INORGCHEM.3C02087.

(3) Stanger, A. Nucleus-Independent Chemical Shifts (NICS): Distance Dependence and Revised Criteria for Aromaticity and Antiaromaticity. *J. Org. Chem.* **2006**, *71* (3), 883–893. https://doi.org/10.1021/JO051746.

(4) Gershoni-Poranne, R.; Stanger, A. The NICS-XY-Scan: Identification of Local and Global Ring Currents in Multi-Ring Systems. *Chem. - A Eur. J.* **2014**, *20* (19), 5673–5688. https://doi.org/10.1002/CHEM.201304307.

(5) Herges, R.; Geuenich, D. Delocalization of Electrons in Molecules. *J. Phys. Chem. A* **2001**, *105* (13), 3214–3220. https://doi.org/10.1021/JP0034426.

(6) Geuenich, D.; Hess, K.; Köhler, F.; Herges, R. Anisotropy of the Induced Current Density (ACID), a General Method to Quantify and Visualize Electronic Delocalization. *Chem. Rev.* **2005**, *105* (10), 3758–3772. https://doi.org/10.1021/CR0300901.

(7) Frisch, M. J.; Trucks, G. W.; Schlegel, H. B.; Scuseria, G. E.; Robb, M. A.; Cheeseman, J. R. Scalmani, G.; Barone, V.; Petersson, G. A.; Nakatsuji, H.; Li, X.; Caricato, M.; Marenich, A. V.; Bloino, J.; Janesko, B. G.; Gomperts, R.; Mennucci, B.; Hratchian, H. P.; Ortiz, J. V.; Izmaylov, A. F.; Sonnenberg, J. L.; Williams-Young, D.; Ding, F.; Lipparini, F.; Egidi, F.; Goings, J.; Peng, B.; Petrone, A.; Henderson, T.; Ranasinghe, D.; Zakrzewski, V. G.; Gao, J.; Rega, N.; Zheng, G.; Liang, W.; Hada, M.; Ehara, M.; Toyota, K.; Fukuda, R.; Hasegawa, J.; Ishida, M.; Nakajima, T.; Honda, Y.; Kitao, O.; Nakai, H.; Vreven, T.; Throssell, K.; Montgomery, J. A., J.; Peralta, J. E.; Ogliaro, F.; Bearpark, M. J.; Heyd, J. J.; Brothers, E. N.; Kudin, K. N.; Staroverov, V. N.; Keith, T. A.; Kobayashi, R.; Normand, J.; Raghavachari, K.; Rendell, A. P.; Burant, J. C.; Iyengar, S. S.; Tomasi, J.; Cossi, M.; Millam, J. M.; Klene, M.; Adamo, C.; Cammi, R.; Ochterski, J. W.; Martin, R. L.; Morokuma, K.; Farkas, O.; Foresman, J. B.; Fox, D. J. Gaussian 16, Revision C.01. Gaussian Inc.: Wallingford CT 2016.

(8) Dirac, P. A. M. Quantum Mechanics of Many-Electron Systems. *Proc. R. Soc. London. Ser. A, Contain. Pap. a Math. Phys. Character* **1929**, *123* (792), 714–733. https://doi.org/10.1098/RSPA.1929.0094.

(9) Slater, J. C. A Simplification of the Hartree-Fock Method. *Phys. Rev.* **1951**, *81* (3), 385. https://doi.org/10.1103/PhysRev.81.385.

(10) Becke, A. D. Density-Functional Exchange-Energy Approximation with Correct Asymptotic Behavior. *Phys. Rev. A* **1988**, *38* (6), 3098. https://doi.org/10.1103/PhysRevA.38.3098.

(11) Lee, C.; Yang, W.; Parr, R. G. Development of the Colle-Salvetti Correlation-Energy Formula into a Functional of the Electron Density. *Phys. Rev. B* **1988**, *37* (2), 785. https://doi.org/10.1103/PhysRevB.37.785.

(12) Becke, A. D. Density-functional Thermochemistry. III. The Role of Exact Exchange. *J. Chem. Phys.* **1993**, *98* (7), 5648–5652. https://doi.org/10.1063/1.464913.

(13) Deglmann, P.; Furche, F.; Ahlrichs, R. An Efficient Implementation of Second Analytical Derivatives for Density Functional Methods. *Chem. Phys. Lett.* **2002**, *362* (5–6), 511–518. https://doi.org/10.1016/S0009-2614(02)01084-9.

(14) Deglmann, P.; Furche, F. Efficient Characterization of Stationary Points on Potential Energy Surfaces. *J. Chem. Phys.* **2002**, *117* (21), 9535–9538. https://doi.org/10.1063/1.1523393.

(15) Kratochvil, B.; Lorah, E.; Garber, C. Silver-Silver Nitrate Couple as Reference Electrode in Acetonitrile. *Anal. Chem.* **1969**, *41* (13), 1793–1796. https://doi.org/10.1021/AC60282A011.
